# Development and validation of children's mind wandering scales

**DOI:** 10.3389/fpubh.2022.1054023

**Published:** 2022-12-07

**Authors:** Zhiwei Cao, Ying Huang, Xiaolan Song, Qun Ye

**Affiliations:** ^1^Department of Psychology, College of Teacher Education, Zhejiang Normal University, Jinhua, China; ^2^School of Pharmacy & School of Biological and Food Engineering, Changzhou University, Changzhou, China; ^3^Key Laboratory of Intelligent Education Technology and Application of Zhejiang Province, Zhejiang Normal University, Jinhua, China

**Keywords:** mind wandering, task-unrelated thoughts, self-generated thoughts, attentional failure, spontaneous thinking, context

## Abstract

**Introduction:**

Mind wandering is generally considered an endogenous mental state that arises spontaneously, which is one of the most common experiences of consciousness and typically occurs at a significant cost to mental health and behavioral performance. Previous studies have shown that mind wandering appears to be a stable trait and can be assessed reliably in adults. Surprisingly little, however, is known about how to measure the frequency of mind wandering in children, given that children can accurately introspect their experiences. The present studies aimed to develop the Frequency of Children's Mind Wandering Scale (CMWS-F) and the Context of Children's Mind Wandering Scale (CMWS-C) to assess the frequency of mind wandering and contexts in which mind wandering occurs for children aged 8 to 11 years.

**Methods:**

The exploratory factor analysis (EFA) and confirmatory factor analysis (CFA) were used to develop the CMWS-F and CMWS-C. To further assess the validity of the scales, we compared the scores in CMWS-F/CMWS-C and the frequencies of probe-caught mind wandering in the typical tasks.

**Results:**

In study 1a, the EFA (*n* = 292) and CFA (*n* = 346) showed that attentional failure and spontaneous thinking were the two main dimensions of CMWS-F. In study 1b, contexts about mind wandering in children could be divided into high-demand and low-demand contexts using EFA (*n* = 258) and CFA (*n* = 347). Study 2 showed moderate positive correlations between the frequencies of probe-caught mind wandering in the tasks and the scores in the scales.

**Discussion:**

The results showed that scores on the two scales could predict the performance on the experimental tasks and further demonstrated empirical validity of the CMWS-F and CMWS-C scales. Taken together, the results of the current studies provided preliminary evidence for the validity and reliability of CMWS-F and CMWS-C in children, which can be used as a reference to balance its downsides and productive aspects of mind wandering.

## Introduction

Mind wandering emphasizes a state of consciousness that arises spontaneously in the waking state, when the content of an individual's consciousness is not determined by subjective will but is occupied by endogenous mental representations; this state can occur either in goal-directed tasks or in resting states (1). Adult studies have found that the frequency of mind wandering in daily life is as high as 46.9%, and at least 30% of the sample answered that mind wandering occurs in almost all activities (2). Furthermore, the frequency of mind wandering in children has also been found to reach 20 to 33% (3). Mind wandering as a trait has significant externalities in individual differences (1). For instance, individuals with a low frequency of mind wandering show that they are focused and have good control over their thinking activities, while individuals with a high frequency show that they are often distracted during the task and cannot maintain coherent thinking, thus affecting the performance of the task. In addition, the association between the frequency of mind wandering and individuals with cognitive or emotional disorders is also receiving increasing attention (4). Thus, it is of great theoretical and practical importance to study mind wandering from the perspective of individual difference (5).

Mind wandering has been extensively studied in adults (6–8), but relatively limited research has been conducted in children (3, 9–13). In fact, mind wandering has been shown to be reliably measured in children (9), and the study of its functions will help to understand the role it plays in children's cognitive and social development. In the present paper, we aim to investigate the characteristics of children's mind wandering by developing psychometric scales (Study 1a and Study 1b) and applying experience sampling method in the laboratory tasks (Study 2). In the section that follow, we review recent advances on theoretical basis and related scales of mind wandering.

Current Concerns Theory tells that once a goal is established, it becomes a current concern event (14, 15), and the individual's cognitive system maintains a high level of accessibility to environmental cues that make it easy to accomplish the goal, and facilitates behavior by enhancing the accessibility of goal-related stimuli. This accessibility will continue until the goal is achieved or abandoned. Assuming that an individual's goals have a hierarchical structure, mind wandering may arise spontaneously because alternative implicit goals are automatically activated, only that the individual fails to realize it at the time. 1 thus argue that mind wandering can be incorporated into the executive control model of attention, treating the occurrence of mind wandering as goal-driven processing that simply shifts executive control from the task at hand to the processing of internal goals, and arguing that this process is cognitively resource-intensive, which further leads to the Perceptual Decoupling Hypothesis (1, 16, 17). Several studies of event-related potentials (ERP) provide support for this hypothesis. Some studies found that participants' P300 amplitude during self-reported mind wandering was reduced relative to the events in the task (6, 18–20). Because the P300 can be treated as an index of executive resources, the decreased P300 amplitude during mind wandering indicates that executive resources have been withdrawn, at least partly, from the primary task and are presumably directed toward task-unrelated thoughts (21).

However, executive failure hypothesis argues that mind wandering arises as a result of executive control failure and that this process is not cognitively resource-intensive (22–24). First, it is the default state to constantly assess the gap between the ideal state and the present state and thus to continuously generate spontaneous thoughts beyond the level of awareness. Second, mind wandering arises during the task when controlled processing is not sufficient to handle the interference generated by spontaneous thought. Studies have found that the default mode network (DMN) and executive control network (ECN) are associated with mind wandering in adults. For instance, neuroimaging studies have shown that the DMN and ECN are activated during mind wandering, and the neural activations in both networks are strongest when subjects lack meta-awareness (25–27). Similarly, evidences suggest that mind wandering in children is related to specific executive functions like inhibition and set shifting/switching (3 which could be due to dysregulation of specific brain regions, like DMN. Recent studies on healthy individuals have shown how dysregulation of action control and inhibition capacity impairs task performance that involves inhibition of actions, indicating that children may have difficulty in switching off the DMN during some tedious tasks which require focused attention and inhibit task-unrelated thoughts (28, 29).

As can be seen from the above theories, researchers have accepted that mind wandering was determined by a combination of automatically generated thoughts in response to environmental or mental cues and the ability of the executive control system to deal with disturbing thoughts (1, 24), but most of the existing scales have been designed based on one perspective only, either focusing on the individual's ability to generate spontaneous thoughts or on the individual's ability to execute control during the task, thus lacking comprehensiveness. Study1a aims to explore the dimension of the generation of children's mind wandering, and we hypothesize that both the spontaneity of individual thinking and the executive control processes are involved from the perspective of scale development.

Based on the opposing hypotheses that executive control inhibits the occurrence of mind wandering and executive control supports the continuity of mind wandering, several studies have shown that individuals with high working memory capacity (WMC) report less mind wandering in the sustained attention response task (SART) (22, 30), reading task (23, 31), and memory span task (32–34) relative to individuals with low WMC, and that individuals with high WMC also report less mind wandering when they are more focused on the task using experience sampling in everyday life (35). In contrast, some studies have shown that individuals with high WMC report more mind wandering than those with low WMC in low cognitive demand visual search tasks and respiratory perception tasks (36), and similar findings have been found in choice reaction time tasks (37), where individuals with high WMC report more mind wandering about future directions. More interestingly, in these studies, when the nature of the task was divided in a different way, WMC did not predict the frequency of mind wandering, e.g., in the above-mentioned experience sampling method (35), there was no association between WMC and mind wandering frequency when the task was divided in terms of whether it was challenging or the level of effort exerted. In addition, no association was found between WMC and mind wandering frequency in high cognitive demand visual search task (36). Indeed, researchers have suggested that individuals' executive control not only inhibits the generation of mind wanderings, but also flexibly adjusts the frequency of mind wanderings according to the demands of the task load (38). Thus, Study 1b aims to explore the dimension of the generation of children's mind wandering from the context of cognitive demand.

Researchers have largely acknowledged that differences in the content and frequency of mind wandering often reflect inter-individual differences in trait level, yet there still lacks validated scale to measure the frequency of mind wandering in children. Through extensive reading of relevant literature, we found that the existing measurement tools have the following drawbacks.

First, some studies have used concepts such as daydreaming and task-unrelated thinking, which are close to mind wandering, as alternatives to study the characteristics of mind wandering in terms of form, content, and frequency. Such studies often change the connotation and extension of the concept of mind wandering, which not only lacks face validity but also makes the survey less relevant [e.g., Imaginal Processes Inventory Questionnaire, (39)].

Second, attentional failure and endogenous spontaneity as the two main features of mind wandering are often taken in specific research contexts. Some scales focus only on the interference of mind wandering with specific tasks, emphasizing the characteristics of mind wandering that are not controlled by the subjective will of individuals. These instruments include Attention Related Cognitive Errors scale (40), Cognitive Failure Questionnaire (41) and Memory Failure Scale (42), which are often used to indirectly account for the frequency of mind wandering. This type of scale focuses on the consequences of attention lapses and correlates well with behavioral task performance, and is often used as a tool to illustrate the undesirable consequences of mind wandering. However, not all mind wandering is unhelpful, and not all lapses are necessarily due to mind wandering, and researchers has also found positive implications for the existence of mind wandering (43, 44). The other part of the scale focuses on the content of spontaneously generated mental activities, such as resting state thought activities. These instruments include Automatic Thought Questionnaire (45) and Resting State Questionnaire (46). On the one hand, these scales were developed based on the content of mind activity, not just the frequency of occurrence. On the other hand, these scales focused on the spontaneity of mind activity, ignoring the important role played by attentional control.

Last but not the least, some researchers have used Mindful Attention Awareness Scale (MAAS), developed by Brown and Ryan (47), to indirectly explain the individual differences on mind wandering, based on the logic that mindfulness and mind wandering are opposing concepts. Although the close conceptual relationship between mindfulness and wind wandering, the strength of their association has been surprisingly low (48). In fact, mindfulness is considered to be a general personality trait, but mind wandering is a much more transient and fluctuating phenomenon during an ongoing task. Thus, the MAAS can be used as a reference to study an individual's level of mind wandering, but is not a substitute.

To sum up, there is no scale developed for children on the frequency of mind wandering. However, elementary school is a critical period for good behavior and a foundation for future learning development, so it is necessary to accurately screen children with excessive mind wandering and lack of mind wandering. For children with excessive mind wandering, early intervention would improve academic performance and quality of life; for children with a lack of mind wandering, a package of counseling exercises would help improve individual attention, and social and emotional functioning.

## Study 1a development of the frequency of children's mind wandering scale (CMWS-F)

### Item generation

We first invited 22 elementary school students for interviews, which included conceptual understanding of mind wandering and frequency of mind wandering. After that, a total of 21 relevant items were generated by combining previous relevant scales, expert opinions, and interview contents.

In order to determine the validity of the generated items, two pilot tests were conducted. The total sample included 220 children, with 94 children in the first polit test (36 boys and 58 girls) and 126 in the second test (59 boys and 67 girls). All participants are elementary school students from grades 3 to 6 in Zhejiang Province, China. The first pilot analysis revealed that there were problems such as inappropriate reverse questions, and low item-total correlations for some items. After the modification, we decided not to apply the reverse questions and replaced the abstract concepts with life-like terms. To explore the validity of the changes, the revised scale was tested for the second time. The item analysis was good and resulted in a preliminary scale. Finally, a total of 22 items were identified (including one lie detection item, e.g., please check option 4 on this question), and all response options were on a 5-point Likert response scale (1 = strongly disagree, 2 = disagree, 3 = unsure, 4 = agree, and 5 = strongly agree).

### Exploratory factor analysis (EFA)

#### Participants

The sample consisted of 332 children, of which 40 children were excluded due to randomly check and failed the lie detection item. The remaining 292 participants' data were analyzed (151 boys and 141 girls, *M* = 10.70 years old, *SD* = 1.30, 24.3% of third graders, 28.1% of fourth graders, 22.6% of fifth graders, 25.0% of sixth graders).

#### Results

The structure of CMWS-F was analyzed using principal component analysis (PCA) and Promax rotation method, and the specific statistical processing was implemented with SPSS 10.0. We first conducted item analysis to assess the item appropriateness within the scale. Inappropriate items were eliminated based on the critical ratio (CR ≥ 3), the item-total correlation (*r* ≥ 0.3). One item was removed first as it did not meet above criteria. One main step before conducting EFA was evaluating the data appropriateness for Factor analysis using Kaiser Meyer-Olkin (KMO) and the Bartlett test of sphericity. The KMO value was 0.926, indicating that the sample size was adequate for factor analysis. The Bartlett test was significant (*p* < 0.001), supporting the argument that the data were appropriate for conducting EFA. Following the EFA, eight items were removed from the scale as their factor loadings fell below 0.45. The scree plot and eigenvalues <1.0 were evaluated to determine the number of factors in this scale. In the end, two factors were extracted and the two factors accounted for 56.32% of the total variance. The two factors respectively accounted for 42.42 and 13.91% of the variance. Factor loadings ranged from 0.59 to 0.84 (see details in Table 1).

**Table 1:** Factor loadings of the CMWS-F.

**Items**	**Factor loadings**	
	**F1 (Attentional failure)**	**F2 (Spontaneous thinking)**
1. I often get distracted when I'm doing something, and I think about other things without realizing it.	**0.807**	0.010
2. During class, I often lose focus and think about other things.	**0.790**	−0.078
3. I would try very hard to listen to the lecture or concentrate on something, but often I would still be thinking about something unrelated to the class.	**0.763**	0.000
4. While thinking about the problem, I often suddenly think of other things that are unrelated to the problem.	**0.742**	0.083
5. I often can't help but think about other interesting things when I'm doing my homework.	**0.735**	−0.004
6. I often do one thing but can't help thinking about another.	**0.616**	0.113
7. I am a person with a lot of ideas, and often suddenly appear some interesting ideas.	−0.225	**0.840**
8. My mind often comes up with one different thought (idea) after another.	0.013	**0.778**
9. I often can't help but make a lot of associations.	0.007	**0.775**
10. I always think about many things without realizing it.	0.219	**0.621**
11. I often have a bee in my bonnet and come up with a lot of ideas without realizing it.	0.141	**0.595**
12. Ideas often pop up in my head for no apparent reason.	0.223	**0.587**

CMWS-F, Frequency of Children's Mind Wandering Scale. The bold values indicate the strongest loading for each item.

Factor 1 consisted of six items in which children frequently “absent-minded” during the process that required them to continuously devote attentional resources on the task at hand. We named this factor “attentional failure.” In contrast, Factor 2 consisted of 6 items that include involuntary thoughts unrelated to the current activity, such as reflections on past events, plans for the future, or even “whimsical thoughts,” which we named “spontaneous thinking.” The correlation coefficient between the two factors was 0.442, indicating the homogeneity and relative independence of the content measured by each dimension.

### Confirmatory factor analysis (CFA)

#### Participants

The sample consisted of 346 children (155 boys, 190 girls, 1 other; *M* = 10.92 years old, *SD* = 1.20, 21.4% of third graders, 25.1% of fourth graders, 35.3% of fifth graders, 18.2% of sixth graders).

#### Results

Considering that mind wandering might differ in frequency for boys and girls, we conducted a series of independent-sample *t*-tests and found that there were no gender differences for each dimension in our scales (*p* > 0.05, Table 2).

**Table 2:** Gender comparisons for CMWS-F/CMWS-C scores in study 1.

**Scale**	**Boys (M ±SD)**	**Girls (M ±SD)**	**t**	**df**	**Cohen'd**
**CMWS-F**					
Attentional failure	2.69 ± 0.96	2.66 ± 0.86	0.352	343	0.03
Spontaneous thinking	3.35 ± 0.89	3.33 ± 0.83	0.262	343	0.02
Total score	3.02 ± 0.81	2.99 ± 0.72	0.356	343	0.04
**CMWS-C**					
High-demand	2.45 ± 0.87	2.40 ± 0.85	0.614	340	0.06
Low-demand	3.34 ± 0.83	3.34 ± 0.79	−0.072	340	0.00
Total score	2.90 ± 0.72	2.87 ± 0.67	0.338	340	0.04

CMWS-F, Frequency of Children's Mind Wandering Scale; CMWS-C, Context of Children's Mind Wandering Scale.

We then used the confirmatory factor analysis (CFA) and test-retest reliability to evaluate the scale. CFA is applied as a way to test the construct's dimensionality and to confirm the factor structure that emerged in the EFA. CFA was conducted using AMOS 4.0, and maximum likelihood estimation (MLE) was used to examine the fit of the model to the data. Evaluation of different indices has been suggested to check the fit of the model. The result revealed that the model provided an acceptable fit to the data, and the factorial structure of children's mind wandering frequency was confirmed (χ^2^/*df* = 3.14, GFI = 0.922, CFI = 0.919, TLI = 0.899, RMSEA = 0.079). All factor loadings were significant at *p* < 0.05.

### Test-retest reliability

Test-retest reliability reflects the stability and consistency of the scale across time. To test the degree of consistency of the scale, we assessed test-retest reliability, and the interval between the retests was 2 weeks. A total of 99 participants completed the scale twice (41 boys and 58 girls, *M* = 10.54 years old, *SD* = 1.08, 25.3% of third graders, 37.4% of fourth graders, 37.4% of fifth graders). The correlation analysis showed that the correlation coefficient of the attentional failure dimension was 0.76 (*p* < 0.01), the correlation coefficient of the spontaneous thinking dimension was 0.74 (*p* < 0.01), and the correlation coefficient of the total score was 0.80 (*p* < 0.01). The results showed that this scale had high test-retest reliability over time.

### Discussion

In this study, the construct validity of the scale was verified in detail in two aspects: overall model fit, and intrinsic structure of the model, and it can be concluded that the two-factor structure of the Frequency of Children's Mind Wandering Scale was supported by the data, and the test-retest reliability of the scale is robust.

Early scholars believed that mind wandering arises simply as a result of failures of attentional control, and thus individual differences in attentional control could predict the frequency of mind wandering. According to this conjecture, adolescents have significantly weaker attentional control than adults, and thus adolescents should experience more mind wandering than adults. Young adolescents, however, did not report more mind wandering than adults (49). Study 1a suggests that mind wandering is the result of both attentional failure and spontaneous thinking, reflecting the individual's executive control and spontaneity of thought, respectively, which we should consider when predicting the frequency of mind wandering. It has been found that the generation of mind wandering is complex in different contexts (50), and the weighting of attentional failure and spontaneous thinking is constantly changing. For this reason, we designed Study 1b to develop a contextual scale for the occurrence of children's mind wandering and to explore how the frequency of children's mind wandering changes in different contexts.

## Study 1b development of the context of children's mind wandering scale (CMWS-C)

The development of the Context of Children's Mind Wandering Scale followed the same four steps of interview and pilot test, exploratory factor analysis, confirmatory factor analysis, and test-retest analysis, each of which was strictly implemented in accordance with psychometric requirements.

### Item generation

The whole process of items generation was similar to the development of the CMWS-F. The total sample included 218 children, with a total of 92 children in the first pilot test and 126 in the second test. Through two rounds of test, a total of 22 items (including one lie detection item) were formed for the initial scale.

### Exploratory factor analysis (EFA)

#### Participants

The sample consisted of 332 children, of which 74 children were excluded due to randomly check and failed the lie detection item. The remaining 258 participants' data were analyzed (134 boys and 124 girls, *M* = 10.71 years old, *SD* = 1.28, 25.6% of third graders, 26.7% of fourth graders, 22.1% of fifth graders, 25.6% of sixth graders).

#### Results

The structure of the CMWS-C was analyzed using PCA and Varimax rotation method, and the specific statistical processing was implemented using SPSS 10.0. Without the lie detection item, we conducted EFA on 21 items. The KMO value was 0.870, indicating that the sample size was adequate for factor analysis. The Bartlett test was significant (*p* < 0.001), supporting the argument that the data were appropriate for conducting EFA. Following the EFA, nine items were removed from the scale as their factor loadings fell below 0.45. Based on the results and scree plot, two factors were extracted. The results showed that the two factors accounted for 51.57% of the total variance. The two factors respectively accounted for 27.98 and 23.59% of the variance. Factor loadings ranged from 0.59 to 0.79 (see details in Table 3).

**Table 3:** Factor loadings of the CMWS-C.

**Items**	**Factor loadings**	
	**Factor 1(high-demand)**	**Factor 2(low-demand)**
1. When I'm doing my homework, I would often be miles away.	**0.791**	0.006
2. Even though I'm concentrating on one thing, I still often get distracted and think about other things.	**0.774**	0.120
3. I often get distracted during class and think about other things.	**0.754**	0.091
4. When I am in the hobby class, I'm often miles away and think about something else.	**0.737**	0.271
5. During the exam, I am sometimes interrupted by other ideas that pop up.	**0.622**	0.082
6. I often get distracted and think of other things while reading.	**0.586**	0.205
7. On the way home by car, I often can't help but think about many things.	0.009	**0.735**
8. When I take a leisurely walk, I often can't help but think about other things.	0.109	**0.725**
9. When I sit alone in the chair resting, I can't help but think of one thing after another.	0.050	**0.684**
10. Before sleeping at night (when you're lying in the bed ready to sleep), things come to my mind.	0.164	**0.631**
11. When I'm brushing my teeth or taking a shower, I can't help but think of other things.	0.181	**0.628**
12. When I finish the textbook that the teacher asks us to finish before the specified time, I can't help but think about something else.	0.464	**0.599**

CMWS-C, Frequency of Children's Mind Wandering Scale. The bold values indicate the strongest loading for each item.

Factor 1 consisted of 6 items across which children's mind wandering invaded in contexts such as reading and taking classes, and thus we named this factor “high-demand.” In contrast, Factor 2 consisted of 6 items across which children's mind wandering happened in contexts such as riding in the car and going for a walk, and this is usually a context where children are prone to mind wandering, and we named this factor “low-demand.”

### Confirmatory factor analysis (CFA)

#### Participants

The sample consisted of 347 children (155 boys,191 girls, 1 other; *M* = 10.92 years old, *SD* = 1.21, 21.6% of third graders, 25.1% of fourth graders, 35.2% of fifth graders, 18.2% of sixth graders).

#### Results

The result revealed that the model provided an acceptable fit to the data, and the factorial structure of CMWS-C was confirmed (χ^2^/*df* = 2.428, GFI = 0.940, CFI = 0.929, TLI = 0.911, RMSEA = 0.064).

### Test-retest reliability

A total of 100 participants completed the scale twice (42 boys and 58 girls, *M* = 10.52 years old, *SD* = 1.09, 26.0% of third graders, 37.0% of fourth graders, 37.0% of fifth graders), and the test-retest analysis showed that the correlation coefficient of the dimension of high-demand context was 0.61 (*p* < 0.01); the correlation coefficient of the dimension of low-demand context was 0.62 (*p* < 0.01); and the correlation coefficient of the total score was 0.64 (*p* < 0.01). The results showed that this scale had a relatively high test-retest reliability over time. In addition, a matched samples *t*-test showed that children reported significantly more mind wandering in low-demand contexts (*M* = 3.40, *SD* = 0.84) than in high contexts (*M* = 2.43, *SD* = 0.85), *t* = 18.68, *p* < 0.001.

### Relationship between the CMWS-F and the CMWS-C

After collapsing the above data, a total of 343 children completed both CMWS-F and CMWS-C (21.3% of third graders, 25.4% of fourth graders, 35.3% of fifth graders, 18.1% of sixth graders). To further examine the effects of attentional failure and spontaneous thinking on the frequency of mind wandering in different contexts, the factors of CMWS-C were used as dependent variables and the factors of CMWS-F as predictor variables in regression analysis. As seen in Table 4, both attentional failure (β = 0.194, *p* < 0.01) and spontaneous thinking (β = 0.514, *p* < 0.001) were valid predictors in the low-demand context, and spontaneous thinking had a greater weight in the regression model. In contrast, only the factor of attentional failure (β = 0.782, *p* < 0.001) was a valid predictor in the high-demand context.

**Table 4:** Multiple regression analysis of the CMWS-F on CMWS-C.

**Dependent variable**	**Predictor variables**	***R***	***R^2^***	***F***	**β (Standardized)**	***t***
Low-demand context	regression model	0.632	0.400	113.319^***^		
	Attentional failure				0.194	4.025^**^
	Spontaneous thinking				0.514	10.653^***^
High-demand context	regression model	0.788	0.620	277.701^***^		
	Attentional failure				0.782	20.365^***^
	Spontaneous thinking				0.012	0.313

** *p* < 0.01,

*** *p* < 0.001. CMWS-F, Frequency of Children's Mind Wandering Scale; CMWS-C, Context of Children's Mind Wandering Scale.

### Discussion

Study 1b shows that the occurrence of children's mind wandering can be broadly classified into two types of contexts: low-demand contexts, which are commonly understood to be prone contexts in which individuals often let go of conscious control and allow their thoughts to drift; and high-demand contexts, in which individuals are occasionally distracted by inner thoughts. The competing relationship between mind wandering and task load has been found in adult research, i.e., mind wandering occurs less when the current task places a higher demand on an individual's cognitive resource, and conversely, mind wandering occurs more frequently. Study 1b illustrated by means of a scale that children did report significantly more frequent mind wandering in low-demand contexts than in high-demand contexts.

In combination with Study 1a and Study 1b, we investigated the psychometric properties of the CMWS-F and CMWS-C. However, it is the first time to develop a scale for children's mind wandering, and it is difficult to find an authoritative method as a calibration for this study. For this reason, we designed Study 2 to support the validity of the scales with more objective behavioral experiments. On the one hand, we can explore the characteristics of children's mind wandering in the laboratory tasks, and on the other hand, we can explore the relationship on subjective reports of mind wandering in the tasks and in our scales.

## Study 2 probe-caught mind wandering in the experimental tasks

### Participants

A power analysis was performed on children's data from a similar protocol to estimate the appropriate sample size (12, 13). With an α = 0.05 and power = 80%, we required at least 82 participants to obtain a medium-sized effect (two-tailed, *r* = 0.3) for the validity of the scales in each laboratory task using the experience sampling method. Additional children were recruited in case of potential withdrawal from our tasks (e.g., inability to perform the computerized task). Finally, the total sample consisted of 365 children (all participants were from grades 4–6, 95 children in Breathing task, 105 children in Sustained Attention Response Task, 94 children in Vigilance task and 71 children in one-back task).

### Materials and procedure

In this study, we selected four typical tasks with “thought probe” inserted to study mind wandering.

In the Breathing task, participants needed to wear headphones and sit in front of a computer screen, close their eyes and pay attention to the movement of breath in and out. In addition, a “ding” sound would come from the headphones from time to time, and children needed to open their eyes to answer the questions that appeared on the screen. The question was as follows: what were you thinking before the sound appeared? The answer options were to think about something related to counting breaths (please press “F”) and to think about something unrelated to counting breaths (please press “J”). Following the judgment, they were prompted to close their eyes and count breaths again. During the task, the program presented 8 times of thought probes, and the time interval between two question pages was 25, 35, 45, and 55s. A 40s buffer was set at the beginning of the program, and the entire program lasted approximately 6 min.

In the Vigilance task, there was only one stimulus “+” presented for 0.5s, and the inter-stimuli interval was 1.5, 2.5, 3.5, or 4.5s. Participants were instructed to press a button whenever they saw the “+.” This task lasted 12 min and collected 30 times of thought probes on mind wandering.

In the Sustained Attention Response Task (SART), the stimuli were divided into two categories: white numbers 1–9 (non-targets) and red crosses (targets). Each stimulus presented for 0.5s and the inter-stimulus interval was 2s. Participants were asked to press the space bar to the numbers as fast and accurately as possible and to withhold the response when the red cross was presented. The ratio of the appearance of non-targets to targets was 8:1 (non-targets = 240, targets = 30), and the program would randomly probe 30 times on mind wandering. The entire program was approximately 11 min.

In the one-back task, participants were asked to wear headphones and sit in front of a computer screen. Two stimuli, black numbers 1–9 (non-target) and green question mark (target), were presented at random order, with 2s for non-target and 3s for target on screen, followed by 1s inter-stimulus interval. Participants were asked to determine the parity of the previous number when the green question mark appeared, pressing “F” for odd numbers and “J” for even numbers. Non-target to target ratio was approximately 7:1 (non-targets = 190, targets = 24). Similarly, the program would randomly appear 6 times on mind wandering, with a “ding” in the headset, asking the participants about their state of consciousness at that time. The entire program was approximately 11 min.

It is important to note that we used thought-sampling method to obtain participants' current state of consciousness. To help participants familiar with the procedures, we presented examples of different options to ensure all of them understood the question before the formal tests. At the end, the participants were asked to complete the CMWS-F and the CMWS-C.

### Results

We first calculated the accuracy of one-back task, and the children showed reasonable task performance (*M*_*one*−*back*_ = 0.80, *SD*_*one*−*back*_ = 0.22). We then evaluated how this task performance was affected by probe-caught mind wandering across subjects, and results showed a significant negative correlation between the task accuracy and the frequency of probe-caught mind wandering (calculated as the proportion of task-unrelated thoughts in probes, *M* = 0.37, *SD* = 0.30; *r*_*one*−*back*_ = −0.277, *p* < 0.05), indicating that the participants who reported more mind wandering were associated with poor performance during a high-demand task. In addition to the breathing task (no response required), we also calculated the average coefficient of variability (CV = SD/mean) for the reaction time in our SART and Vigilance tasks (*M*_SART_ = 0.34, *SD*_SART_ = 0.22, *M*_Vigilance_ = 0.38, *SD*_Vigilance_ = 0.22), which is consistent with our previous children's study (13). Taken together, these results suggested that children are able to complete these computerized tasks.

Next, we considered the correspondence between the state-level (probe-caught) and the trait-level mind wandering by calculating the correlation between the frequencies of mind wandering in laboratory tasks and the scores on CMWS-F/CMWS-C. As expected, the results showed moderate positive correlations between the frequencies of probe-caught mind wandering in the three tasks and the scores in the scales, especially in the dimension of attentional failure of CMWS-F, with correlation coefficients ranging from 0.284 to 0.316 (Table 5).

**Table 5:** Correlations between the frequencies of probe-caught mind wandering in tasks and the scores of the CMWS-F/CMWS-C in Study 2 (descriptive statistics included).

**Task Type (probe-caught mind wandering)**	**TUT probability** **(*M* ±*SD*)**	**CMWS-F (*****M*** ±***SD*****)**		**CMWS-C (*****M*** ±***SD*****)**	
		**Attentional failure**	**Spontaneous thinking**	**High-demand**	**Low-demand**
Breathing task	0.43 ± 0.26	15.57 ± 5.08 (0.316^**^)	19.17 ± 5.58 (0.180)	13.86 ± 5.81 (0.236^*^)	19.75 ± 5.37 (0.101)
SART	0.17 ± 0.14	14.65 ± 4.90 (0.318^**^)	18.68 ± 5.70 (0.197^*^)	13.30 ± 5.71 (0.180)	19.35 ± 5.40 (0.164)
Vigilance task	0.14 ± 0.12	14.73 ± 5.01 (0.284^**^)	19.13 ± 5.67 (0.138)	13.70 ± 6.01 (0.186)	19.47 ± 5.08 (0.184)

* *p* < 0.05,

** *p* < 0.01. The data in the parenttheses represented the correlation coefficient between the scores on CMWS-F/CMWS-C and probe-caght mind wandering in tasks. CMWS-F, Frequency of Children's Mind Wandering Scale; CMWS-C, Context of Children's Mind Wandering Scale. The probability of task-unrelated thought (TUT) was calculated as the proportion of task-unrelated thoughts in probes.

### Discussion

Early scholars often wonder when children are able to correctly understand and report conscious state. Studies of young children aged 4 to 13 years suggested that the uncontrollability of comprehension awareness did not mature until 8 or 9 years of age or even later (51–53). Here, we show that children are able to correctly report their mind wandering, at least, at the age of 8 years old. Mind wandering has been shown in numerous studies in adults to be associated with sustained attention capacity (54–57), working memory (23, 30), and intelligence tests (32). Usually when individuals report more mind wandering during the task, the worse the individual performs on the task, and vice versa. Mrazek and colleagues found that the higher the frequency of mind wandering during adolescent reading, the worse the reading comprehension scores, and concluded that students aged 11–13 years already have the ability to correctly report their state of consciousness (5). In all three different task conditions, we derived an agreement between mind wandering frequencies reported in the task and the mind wandering frequencies measured by the scale, confirming the criterion validity of the CMWS-F/CMWS-C.

Study 1a showed that the production of mind wandering was influenced by both attentional failure and spontaneous thinking, while study 1b showed that the contexts in which mind wandering occurred could be broadly classified into two categories: low-demand and high-demand, and the weights of the effects of attentional failure and spontaneous thinking on the frequency of mind wandering changed continuously in different contexts, constituting a dynamic and complex relational model. This would explain why the use of working memory capacity to predict mind wandering frequency under different experimental task conditions has been controversial (17).

## General discussion

This is the first study to develop the CMWS-F and CMWS-C scales to assess the frequency of mind wandering for children. Following the steps of standard scale development (item generation and selection, EFA, CFA and test-retest analysis), we showed scales with good reliability and validity. Study 2 further provided evidence for the criterion validity of the CMWS-F and CMWS-C scales within four laboratory tasks, which showed moderate consistency between the frequencies of probe-caught mind wandering in the tasks and the scores in the scales.

Attentional failure and spontaneous thinking are two main factors that caused mind wandering in the CMWS-F. Attentional failure occurs when an individual's attention shifts from the current task to something unrelated, which arises as a result of executive control failure (22–24). Executive control is closely related to the main characteristic of mind wandering: disengagement of the external task/environment (58). Children's capacity for attentional control is relatively immature throughout childhood and adolescence (59, 60), so their inhibitory control capacity is so weak that they are not able to focus on the current task long time. Therefore, it's not hard to explain why inhibitory control capacity is a significant predictor of children's mind wandering frequency (3).

The second factor, spontaneous thinking, means that individual's mentation is occupied by implicit goals, such as memories from the past and plans for the future [also called unintentional mind wandering, (61)]. On the one hand, studies have shown that spontaneous thinking is correlated with depression, stress and anxiety disorder (62, 63). In terms of personality, Spontaneous thinking has negative correlations with agreeableness, and extraversion (64). Thus, this may also bring negative consequences, like emotional problems and learning difficulties, for children. Therefore, if children score too high on the CMWS-F for spontaneous thinking, parents should pay attention to whether the children have emotional and interpersonal problems. On the other hand, this does not mean spontaneous thinking is useless for children. Studies have shown that mind wandering has a prospective bias (65–68), the content of mind wandering about future planning is significantly more than that about past, suggesting that mind wandering may contribute to the ability of future planning. Indeed, Ye et al. (12) found that there is a significant forward-looking bias of mind wandering among children. All of these suggest that only by correctly understanding the causes and the characteristics of children's mind wandering, can we give full play to the positive role of mind wandering and reasonably explore how to restrain children with high-frequency mind wandering and improve children with low-frequency mind wandering.

The CMWS-C showed that the contexts mind wandering often occurs in children can be divided into two categories: low-demand context and high-demand context. For children, the low-demand context is the leisure context like playing games and having a walk; the high-demand context is the task context like doing homework and reading books. Mind wandering can be beneficial or detrimental depending on the flexibility of cognitive resources in the specific context (58). In easy tasks that only demand low cognitive resources, individuals can tolerate longer delays in waiting for rewards because of mind wandering (69). However, in high cognitively demanding tasks, studies have shown that mind wandering could be detrimental for individual's performance (18, 70–72). This reveals us to the children's mind wandering treatment should also be flexible, in low-demand context, children can be relatively unfettered when mind wandering occurs, while in high-demand context, children must be required to pay attention to the current task.

In study 2, we also found some evidence for CMWS-F and CMWS-C in experimental tasks. There was a significant correlation between the score of attentional failure dimension in CMWS-F and three tasks (*p* < 0.01), but no significant correlation on the spontaneous thinking. As expected, attentional failure and spontaneous thinking occur in different contexts. Because the participants were asked to complete the laboratory tasks during the experiment, they were actually in high-demand context. Just like our definitions of the two dimensions of CMWS-F, attentional failure occurs more during the task, while spontaneous thinking occurs more when the individual is free or in low-demand context. This can also explain why the scale score is significantly or nearly significantly correlated with the three tasks under the high-demand dimension in CMWS-C (*p* = 0.07).

As mentioned above, mind wandering has both benefits and costs (43), so it should be viewed dialectically in the field of education. Under some conditions, mind wandering will promote students' creativity (73). But we have to admit that in many educational contexts, mind wandering could lead some negative influence. The higher the mind wandering frequency, the worse the test scores were (74). Besides, if students fail to pay attention to the classroom or what they are learning, this may impede their chances of acquiring important knowledge or skills. The frequency of mind wandering mediates the relation between children's ratings of topic interest and learning scores (9), so improving children's interest in what they have learned is a good choice to counteract the downsides of mind wandering.

Previous studies focused on the influence of divided attention on children from the cognitive process perspective (75), ignoring the spontaneous characteristics of consciousness. Here we showed that spontaneous thinking and attentional failure are the main causes of mind wandering, and discussed the influence of context on children's mind wandering, which will be enlightening for future studies on children's learning difficulties like ADHD (76, 77). Furthermore, meta-awareness hypothesis showed that individuals who are more aware of their current mental activity could have a lower frequency of mind wandering (1, 5). Considering the uncontrollability of children's mental states, one direction for future study is to enhance children's metacognition to indirectly prevent excessive mind wandering. Similarly, mindfulness training, including practices that enhance awareness of thoughts, may modulate the occurrence of mind wandering, supported by bottom-up and top-down neural mechanisms (78).

Finally, it is not certain whether the structure of the CMWS-F/CMWS-C are different in varied cultures. Therefore, more cross-culture studies are needed to explore children's mind wandering. In conclusion, we developed and validated the CMWS-F/CMWS-C scales to explore the underlying causes and contexts of mind wandering in children. The results showed that children's mind wandering was mainly caused by attentional failure and spontaneous thinking, and the contexts could be divided into high-demand context and low-demand context. We have to admit that although our study provided a convenient measurement tool for future studies of mind wandering in children, more research is needed to complement and enrich CMWS-F/CMWS-C.

## Data availability statement

The raw data supporting the conclusions of this article will be made available by the authors, without undue reservation.

## Ethics statement

The studies involving human participants were reviewed and approved by the Ethics Committee of Zhejiang Normal University. Written informed consent to participate in this study was provided by the participants' legal guardian/next of kin.

## Author contributions

XS and QY designed the research. ZC and QY collected the data and performed the statistical analysis. YH and QY wrote the first draft of the manuscript. All authors contributed to the research and approved the final version of the manuscript.
